# Draft Genome Sequence of the Shrimp Pathogen Vibrio parahaemolyticus ST17.P5-S1, Isolated in Peninsular Malaysia

**DOI:** 10.1128/MRA.01053-18

**Published:** 2018-09-20

**Authors:** Sridevi Devadas, Subha Bhassu, Tze Chiew Christie Soo, Fatimah M. Yusoff, Mohamed Shariff

**Affiliations:** a Department of Fisheries Malaysia, Selangor Fisheries Biosecurity Centre, Sepang, Selangor, Malaysia; b Department of Genetics and Molecular Biology, Institute of Biological Sciences, Faculty of Science, University of Malaya, Kuala Lumpur, Malaysia; c Institute of Bioscience, Universiti Putra Malaysia, Serdang, Selangor, Malaysia; d Department of Veterinary Clinical Studies, Faculty of Veterinary Medicine, Universiti Putra Malaysia, Serdang, Selangor, Malaysia; University of Delaware

## Abstract

We sequenced the genome of Vibrio parahaemolyticus strain ST17.P5-S1, isolated from Penaeus vannamei cultured in the east coast of Peninsular Malaysia. The strain contains several antibiotic resistance genes and a plasmid encoding the Photorhabdus insect-related (Pir) toxin-like genes, *pirAvp* and *pirBvp*, associated with acute hepatopancreatic necrosis disease (AHPND).

## ANNOUNCEMENT

Acute hepatopancreatic necrosis disease (AHPND) is a shrimp bacterial disease caused by Vibrio spp. carrying plasmid encoding homologues of the Photorhabdus insect-related (Pir) toxins *pirAvp* and *pirBvp*. The disease can cause up to 100% mortality in farmed penaeid shrimp (1). In addition, the AHPND-causing Vibrio parahaemolyticus (*Vp*_AHPND_) and Vibrio campbellii (*Vc*_AHPND_) also have been discovered carrying tetracycline resistance (2) and multiple antibiotic resistance genes (3), respectively. This study presents the draft genome sequence of a *Vp*_AHPND_ strain, ST17.P5-S1, which was found to contain multiple antibiotic resistance genes. The strain was isolated from the gut of AHPND-affected Penaeus vannamei shrimp farmed in the east coast of Peninsular Malaysia in April 2017. The isolated *Vp*_AHPND_ strain ST17.P5-S1 was deposited at the Universiti Putra Malaysia Institutional Repository (UPMIR), Malaysia, for future investigation.

The genomic DNA of the strain was extracted from an overnight culture grown in tryptic soy broth (TSB) supplemented with 2% sodium chloride (NaCl) using an EasyPure bacterial genomic DNA kit (TransGen, China). Genome sequencing was performed using an Illumina MiSeq sequencer and a MiSeq reagent kit v2 (500 cycles) at Majorbio Bio-Pharm Technology Co., Ltd. (Shanghai, China). The sequence data were assembled using SOAP*denovo* v2.04 and GapCloser v1.12 (4). The assembled contigs were predicted using Glimmer v3.02 (5). The rRNA and tRNA genes were predicted using Barrnap 0.4.2 (6) and the tRNAscan-SE v1.3.1 search server (7), respectively. The data were searched for homologous sequences among several strains using the basic BLAST nucleotide (BLASTn) program.

The genome sequence had 2,327,866 × 2 clean data pair reads (average coverage, 211.91×). The genome was assembled into 130 scaffolds with more than 1,000 base pairs (bp) with an *N*_50_ length of 199,471 bp. The contigs contain 5,125 predicted coding sequences with an average gene length of 891.41 bp, 1 5S rRNA, 2 23S rRNA, and 82 tRNA genes. The 16S rRNA gene was not detected in the assembled genome, probably due to incomplete genome coverage with the sequencing method. However, the strain was detected as positive for the 16S rRNA gene with PCR. The genome of *Vp*_AHPND_ ST17.P5-S1 has an estimated 5,455,642 bp with a G+C content of 45.4%. The assembled genome contained two chromosomes, designated Chr I (∼3,377,580 bp) and Chr II (∼1,908,926 bp), and a plasmid, designated pST17.P5-S1 (∼70 kbp), encoding homologs of the *Photorhabdus* insect-related (Pir) toxins *pirA_vp_* and *pirB_vp_*, which are deadly to shrimp. The genome of the strain also contained phage-related genes, secretion system types II, III, and VI (T2SS, T3SS, and T6SS), and several genes that encode resistance to tetracycline, sulfanomide, bleomycin, acriflavine, bacitracin, chloramphenicol, and vancomycin. The strain also displayed ∼100% similarity with *Vp*_AHPND_ strain NCKU_TV_3HP originating from Thailand (8). This draft genome sequence is essential for better understanding the biodiversity of AHPND and conducting a comparative genomic analysis of AHPND-causing strains in the shrimp industry.

### Data availability.

This whole-genome shotgun project of *Vp*_AHPND_ strain ST17.P5-S1 has been deposited at DDBJ/ENA/GenBank under the accession number PJOR00000000. The version described in this paper is version PJOR01000000.
